# First records of *Pseudodoros nigricollis* Becker (Diptera: Syrphidae) from Cyprus

**DOI:** 10.3897/BDJ.4.e8139

**Published:** 2016-03-28

**Authors:** André van Eck, Christodoulos Makris

**Affiliations:** ‡ Mitox Trial Management B.V., Tilburg, Netherlands; § Unaffiliated, Limasol, Cyprus

**Keywords:** *Hyalopterus pruni*, *Phragmites australis*, distribution, new records, habitat

## Abstract

**Background:**

The hoverfly *Pseudodoros nigricollis* Becker, 1903 is a rarely collected species, of which information on its distribution and ecology is poorly understood.

**New information:**

In this paper the first records of the hoverfly *Pseudodoros nigricollis* from Cyprus are provided and discussed. The discovery indicates that this Afrotropical species is approaching the European continent. Short notes on the habitat in which it has been collected are provided. The relationship with the mealy plum aphid *Hyalopterus pruni* is discussed. Clues on further research are given.

## Introduction

Since it was first described, the hoverfly *Pseudodoros nigricollis* Becker, 1903 has received little attention in the literature. Besides some scattered records, its past and present distribution is poorly known. There are about twenty verified and published specimens from very scattered localities in the Middle East and the eastern half of Africa, ranging from Israel in the north to Madagascar and South Africa in the south (Kassebeer (2000). Furthermore, information on its biology is limited. At present there is no information about its habitat, except for some rearing of larvae apparently found in Egypt on reed-grass (*Phragmites australis*) and on banana leaves (*Musa* sp.) in colonies of the mealy plum aphid *Hyalopterus pruni* (Geoffroy, 1762). In 2014 the second author caught the first specimens of *Pseudodoros nigricollis* in Cyprus.

### General notes on habitat and biology

According to Efflatoun (1922) the two males of *P. nigricollis* were reared, from larvae feeding on the mealy plum aphid *Hyalopterus pruni*' (Geoffroy, 1762) (Homoptera, Aphididae) (published as *Aphis pruni*; source: http://aphid.speciesfile.org/Common/basic/Taxa.aspx?TaxonNameID=1166144), one "on reed-grass" and the other "from banana plant". This aphid has a worldwide distribution, as it is known from Europe, Africa, Asia, North America and Micronesia (Marianas, western Carolines) (Essig 1956, Smith 1936). In the temperate regions it overwinters on many species of *Prunus* (assumed favourably on *P. domestica*) and transfers during the summer to secondary hosts, such as *Arundo*, *Calamagrostis*, *Phalaris*, *Phragmites* (*communis*), *Poa*, *Scirpus*, *Typha* (*latifolia*), and possibly other related plants (Essig 1956, Smith 1936). However some of these secondary hosts are considered doubtful by Smith (1936). Although in the past *P. nigricollis* was only found in association with the mealy plum aphid, its distribution pattern suggests that it is likely that the larvae feed on other aphid species as well. It is interesting to quote Smith 1936 on this issue: "*From a study of the literature the following generalization can be drawn: (1) In the colder regions of the temperate zones* Hyalopterus pruni *attacks plums chiefly or exclusively, and* Phragmites communis *is the usual secondary host. (2) In the warmer temperate and tropical regions* H. pruni *attacks chiefly peach, apricot, and almond, while* Arundo donax *and* Phragmites communis *serve as secondary hosts. This peculiar host specificity, related to climate, is no doubt the result of the importation of the normally plum-feeding aphid into the warmer regions where plums are rare, and finally into the tropics where plums are absent, with the result that the aphid became adapted to peach, apricot, and almond.*" Other than the record by Efflatoun claiming that *P. nigricollis* was "bred" from banana plant, no other sources were found that confirm the presence of the mealy plum aphid on this plant. The only reported aphid on banana is the banana aphid *Pentalonia nigronervosa* Coquerel, 1859, which is present worldwide where banana (*Musa* spp.) is grown (Wright 2006).

## Materials and methods

**Figure 1.**
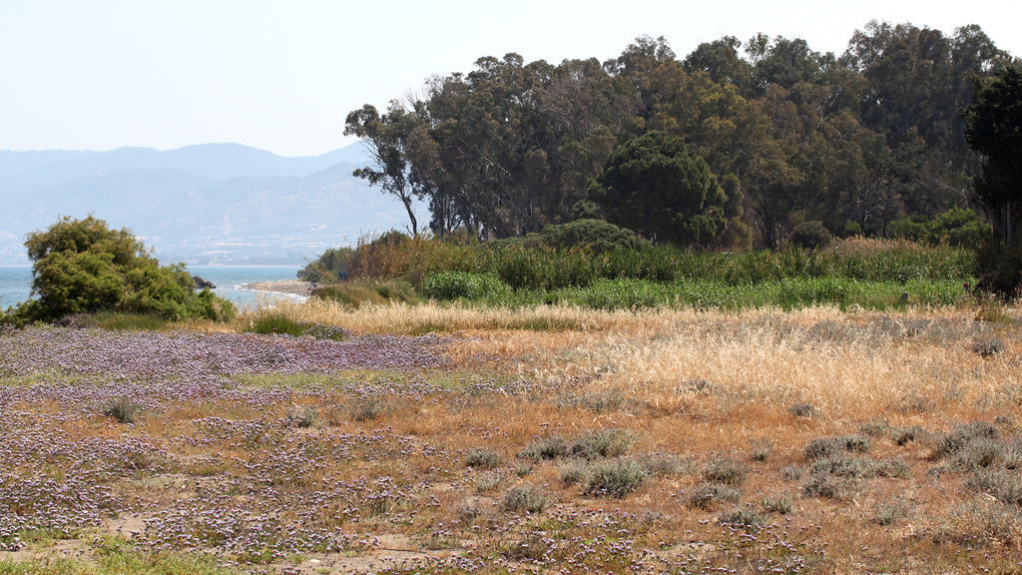
Locality where *P. nigricollis* was collected twice, 9 May 2015. Photo: Christodoulos Makris

**Figure 2.**
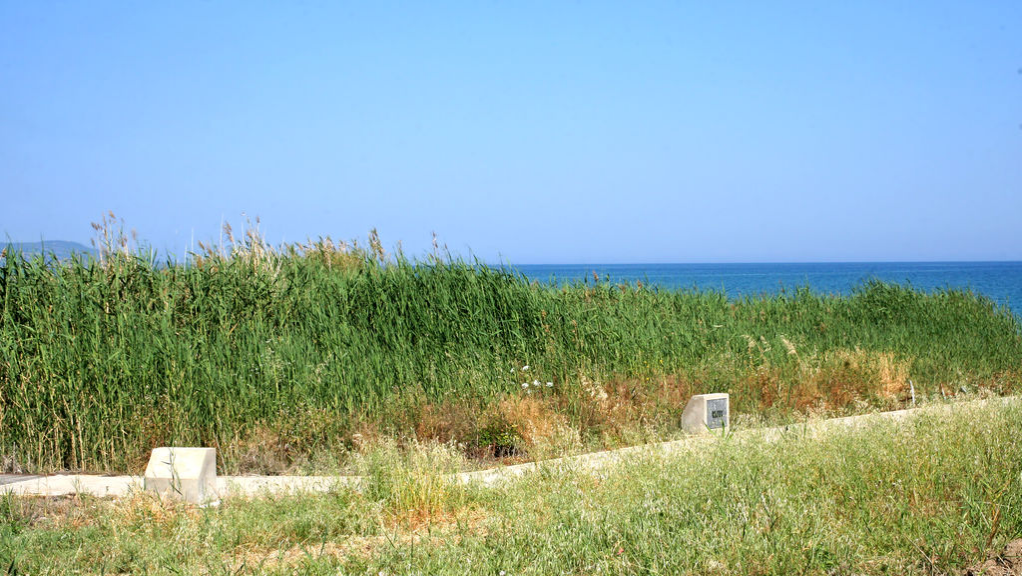
*Phragmites* vegetation where *P. nigricollis* was collected twice, 9 May 2015. Photo: Christodoulos Makris

**Figure 3.**
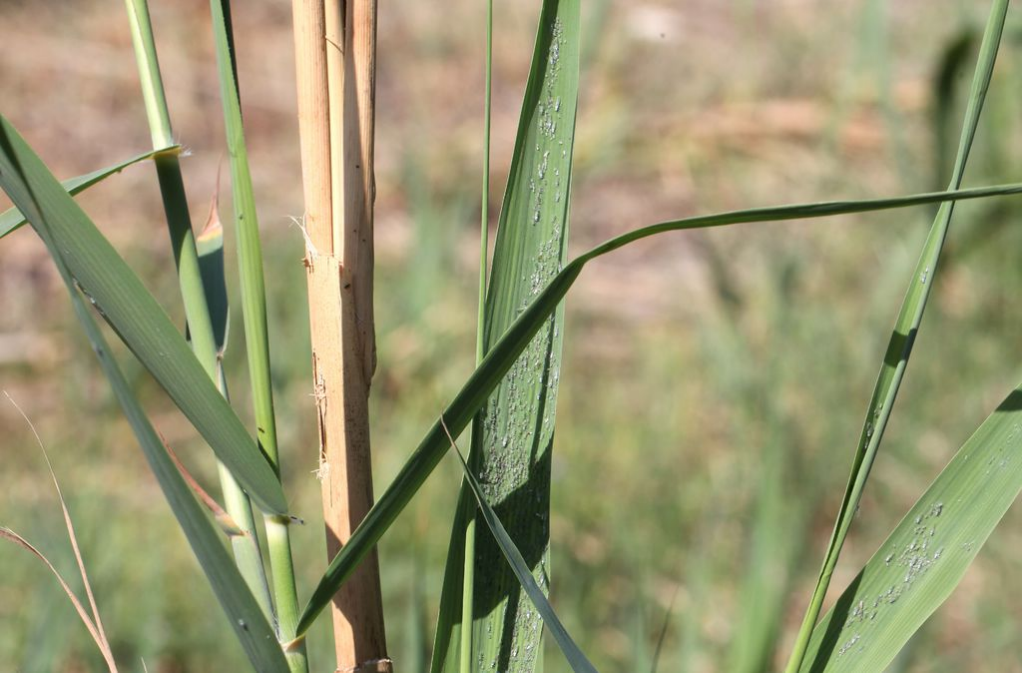
Hyalopterus cf. pruni on *Phragmites australis*. Polis, 9 May 2015. ID J. Prinsen. Photo Christodoulos Makris

**Figure 4.**
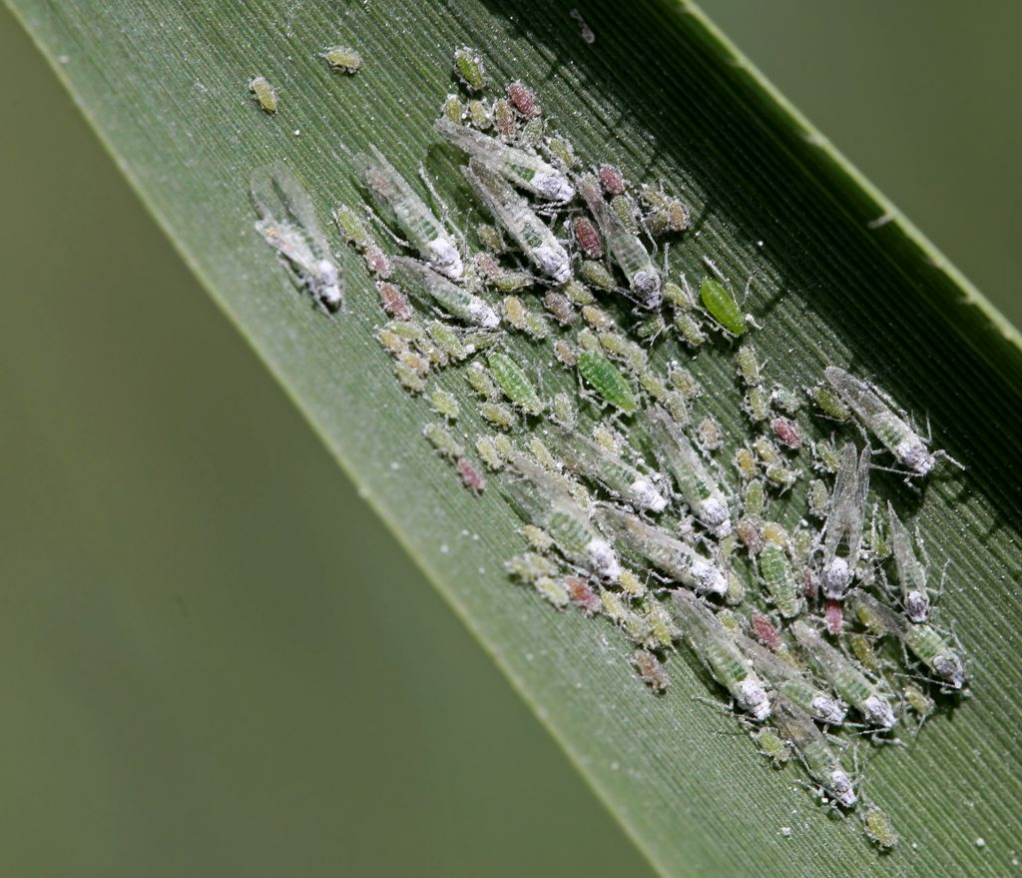
Hyalopterus cf. pruni on *Phragmites australis*. Polis, 9 May 2015. ID J. Prinsen. Photo Christodoulos Makris

**Figure 5.**
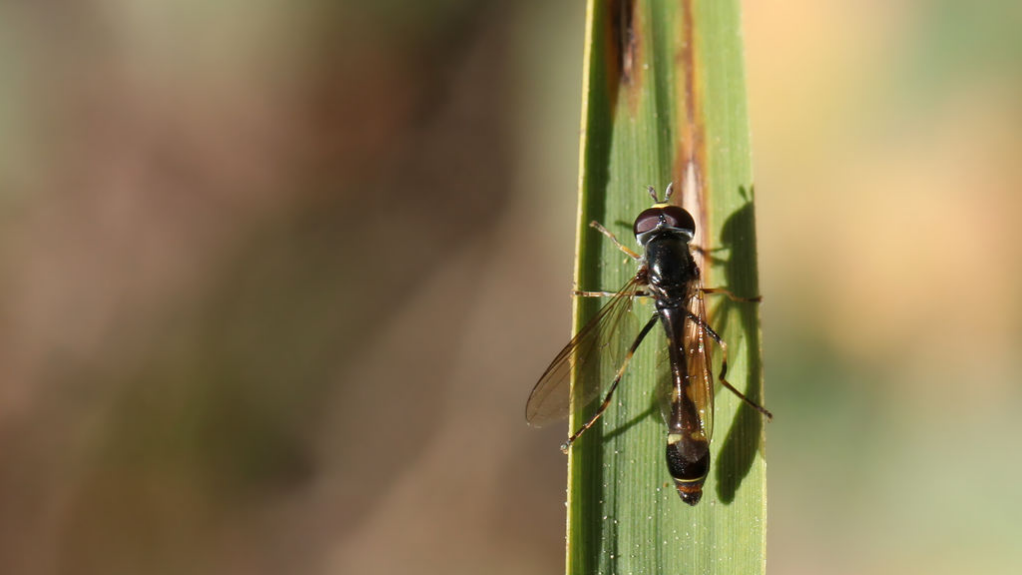
*P. nigricollis* male on 14 November 2015. Photo: Christodoulos Makris

**Figure 6.**
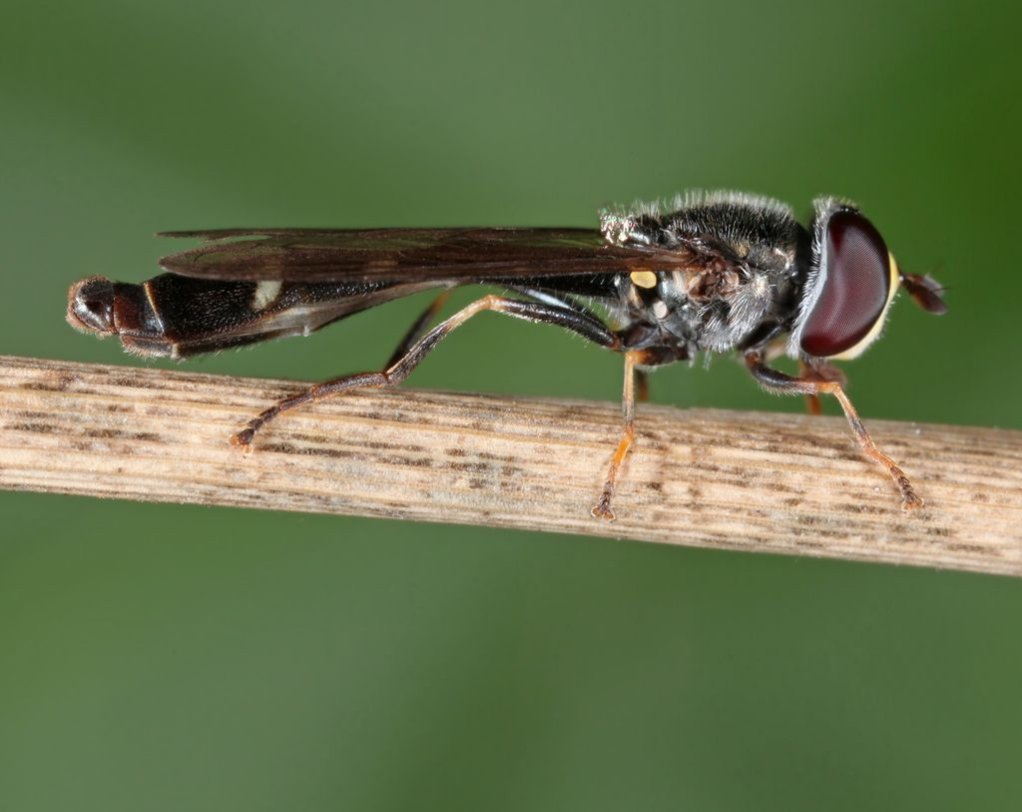
*P. nigricollis* male on 14 November 2015. Photo: Christodoulos Makris

**Figure 7.**
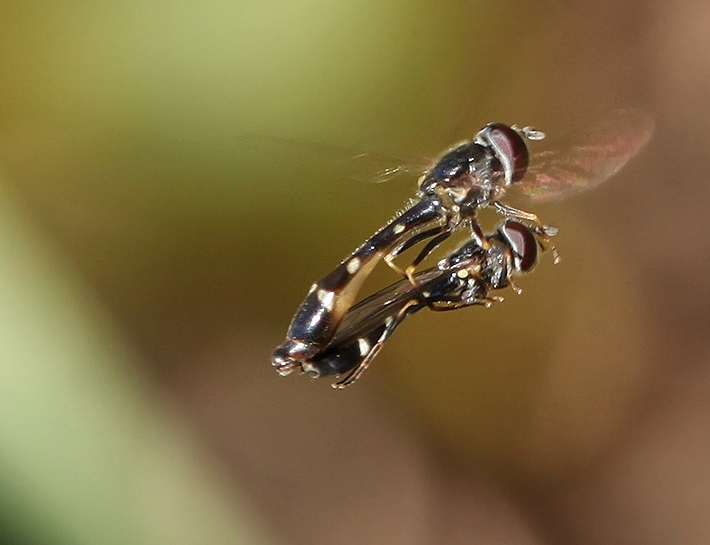
Observed mating in flight on 14 November 2015 of *P. nigricollis*. Photo: Christodoulos Makris

The flies were collected by means of a hand net, in a small reed-bed on the beach west of the village Polis Chrysochou, on the north-west coast of Cyprus. This reed-bed grows in a damp location near the sea and very close to the estuary of the Stavros tis Psokas River (Figs 1, 2). The vegetation is dominated by *Phragmites australis*, *Rubus sanctus* and *Cynanchum acutum*, which is a climber on the reeds, and very rare in Cyprus. The reeds are surrounded by fields, citrus orchards, tourist villas and gardens. There are also extensive reeds along the river and an eucalyptus plantation which is used as a camping site. Searches at the collecting locality in spring 2015 revealed no specimens. On 9 May 2015 aphid colonies were present on *Phragmites* (Figs 3, 4), but the only hoverflies present were *Sphaerophoria scripta* (Linnaeus, 1758). On 14 November 2015, almost one year after the first find, at least fifteen specimens were observed and photographed (Figs 5, 6) and five of them were collected. One pair was observed mating (Fig. 7). All flies were present in an area sheltered from the wind, and all were flying low, near the ground or were resting on the leaves of grasses. One specimen was observed visiting the flowers of *Mercurialis annua* (Euphorbiaceae). Despite an extensive search for aphid colonies on this date none were found. *Pseudodoros nigricollis* has not yet been recorded at other localities in Cyprus.

## Taxon treatments

### Pseudodoros (Pseudodoros) nigricollis

Becker, 1903

http://www.catalogueoflife.org/col/details/species/id/9739bf1196b18500b0e8c8dd4b6bf0bf

Pseudodoros (Pseudodoros) nigricollis The current consensus is that the genus *Pseudodoros* Becker, 1903 consists of two subgenera (*Dioprosopa* Hull, 1947 (Hull 1947) and *Pseudodoros* with three described species: P. (Dioprosopa) clavata (Fabricius, 1794), P. (Dioprosopa) vockerothi (Kassebeer, 2000) and P. (Pseudodoros) nigricollis Becker, 1903. A review of the genus *Pseudodoros* was undertaken by Kassebeer (2000) in which he described a new species from South America and concluded that the subgenus *Dioprosopa* should have generic status with the genus *Pseudodoros* restricted to the Eastern Mediterranean and Afrotropics and *Dioprosopa* to the New World. A fourth species, *P. psyllidivora* Séguy, 1953 was synomymised by Kassebeer (2000) with *Allobaccha sapphirina* (Wiedemann, 1830). Mengual et al. (2008), in their study on the tribe Syrphini, recognised *Dioprosopa* as a subgenus of *Pseudodoros*. The holotype of *P. nigricollis* is a male specimen from Cairo, Egypt, collected by Becker in November 1898. Becker published the description of the genus and species in 1903 (Becker 1903, Becker 1902). Later, Efflatoun (1922) mentioned two males from Ghezireh (which nowadays is part of Cairo), one recorded from spring 1909 and the other without date. Efflatoun's redescription (mostly a direct translation) was based primarily on Becker's description, with additional characters from his two specimens. In his review, Kassebeer (2000) redescribed the male of *P. nigricollis* using the holotype, added further morphological characteristics of the female to those provided for both sexes by Sack (1932) and figured both male and female abdomens. He also agreed with Smith and Vockeroth (1980) that *Baccha extranea* Bezzi 1915 is a junior synonym of *P. nigricollis*. However, he made no reference to Efflatoun's paper or specimens. According to Efflatoun (1922), a male from 1909 was in his private collection (in Cairo) and the other one in the collection of what was called the Sultania Agricultural Society, also based in Cairo. Where these specimens are at present is unknown to the authors. Also it is unknown which females were used by Sack (1932).

#### Materials

- **Type status:** Other material. **Occurrence:** recordedBy: C. Makris; individualCount: 2; sex: males; disposition: in collection A van Eck & C. Makris; **Taxon:** genus: Pseudodoros; specificEpithet: nigricollis; **Location:** locationID: Polis Chrysochou; higherGeographyID: Pafos; country: Cyprus; verbatimElevation: 0 m; verbatimLatitude: 35.04220; verbatimLongitude: 32.41530; verbatimCoordinateSystem: decimal degrees; verbatimSRS: WGS84; **Identification:** identifiedBy: André van Eck; **Event:** samplingProtocol: hand net; eventTime: 11.00-11.30 PM; verbatimEventDate: 30.XI.2014; habitat: *Phragmites australis* reed bed; eventRemarks: sunny day, approx. 19-22 degrees Celcius
- **Type status:** Other material. **Occurrence:** recordedBy: C. Makris; individualCount: 5; disposition: not collected; **Taxon:** genus: Pseudodoros; specificEpithet: nigricollis; **Location:** locationID: Polis Chrysochou; higherGeographyID: Pafos; country: Cyprus; verbatimElevation: 0 m; verbatimLatitude: 35.04220; verbatimLongitude: 32.41530; verbatimCoordinateSystem: decimal degrees; verbatimSRS: WGS84; **Identification:** identifiedBy: André van Eck; **Event:** samplingProtocol: hand net; eventTime: 11.00-11.30 PM; verbatimEventDate: 30.XI.2014; habitat: *Phragmites australis* reed bed; fieldNotes: on and between the reeds in a wind sheltered spot; eventRemarks: sunny day, approx. 19-22 degrees Celcius
- **Type status:** Other material. **Occurrence:** recordedBy: C. Makris; individualCount: 3; sex: males; disposition: in collection A van Eck; **Taxon:** genus: Pseudodoros; specificEpithet: nigricollis; **Location:** locationID: Polis Chrysochou; higherGeographyID: Pafos; country: Cyprus; verbatimElevation: 0 m; verbatimLatitude: 35.04220; verbatimLongitude: 32.41530; verbatimCoordinateSystem: decimal degrees; verbatimSRS: WGS84; **Identification:** identifiedBy: André van Eck; **Event:** samplingProtocol: hand net; eventTime: 09.00-10.30 PM; verbatimEventDate: 14.XI.2015; habitat: *Phragmites australis* reed bed; eventRemarks: sunny day, approx. 21-25 degrees Celcius
- **Type status:** Other material. **Occurrence:** recordedBy: C. Makris; individualCount: 2; sex: 1 male, 1 female; disposition: in collection ZFMK (Bonn, Germany); **Taxon:** genus: Pseudodoros; specificEpithet: nigricollis; **Location:** locationID: Polis Chrysochou; higherGeographyID: Pafos; country: Cyprus; verbatimElevation: 0 m; verbatimLatitude: 35.04220; verbatimLongitude: 32.41530; verbatimCoordinateSystem: decimal degrees; verbatimSRS: WGS84; **Identification:** identifiedBy: André van Eck; **Event:** samplingProtocol: hand net; eventTime: 09.00-10.30 PM; verbatimEventDate: 14.XI.2015; habitat: *Phragmites australis* reed bed; eventRemarks: sunny day, approx. 21-25 degrees Celcius
- **Type status:** Other material. **Occurrence:** recordedBy: C. Makris; individualCount: 10; disposition: not collected; **Taxon:** genus: Pseudodoros; specificEpithet: nigricollis; **Location:** locationID: Polis Chrysochou; higherGeographyID: Pafos; country: Cyprus; verbatimElevation: 0 m; verbatimLatitude: 35.04220; verbatimLongitude: 32.41530; verbatimCoordinateSystem: decimal degrees; verbatimSRS: WGS84; **Identification:** identifiedBy: Christodoulos Makris; **Event:** samplingProtocol: hand net; eventTime: 09.00-10.30 PM; verbatimEventDate: 14.XI.2015; habitat: *Phragmites australis* reed bed; fieldNotes: on and between the reeds in a wind sheltered spot; eventRemarks: sunny day, approx. 21-25 degrees Celcius

#### Description

***Citation*:** Pseudodoros (Pseudodoros) nigricollis Becker, 1903. Becker T (1903) Agyptische Dipteren. Mitteilungen aus dem Zoologischen Museum in Berlin 2, 67-195.

***Differential diagnosis:*** (source: Mengual, X. (2016) Syrphidae Community Website. Accessed at: syrphidae.myspecies.info/taxonomy/term/973/descriptions on 2016-02-19)

*Pseudodoros* is a genus of Syrphini. *P. nigricollis* has eye bare; metasternum bare; thorax without yellow maculae except on scutellum; postmetacoxal bridge incomplete; vein M1 slightly sinuate; vein R4+5 straight or nearly so. The subgenus *Pseudodoros* has abdominal tergum 2 with a median pair of small yellowish maculae; scutellum dark brown; scutum with long, erect pile; pro- and mesotibiae yellow with medial narrow brownish ring; medial black facial vitta narrower than the basoflagellomere's width; mouth edge yellow.

***Description*:** (source: Mengual, X. (2016) Syrphidae Community Website. Accessed at : syrphidae.myspecies.info/taxonomy/term/973/descriptions on 2016-02-19)

##### Male

*Head*: Face with median, small facial tubercle, not produced forward (oral margin less prominent than antennal bases), yellow, oral margin yellow, with medial narrow black vitta, yellow pilose; gena yellow anteriorly, brownish posteriorly, pale pilose; lunule dark, dark area connected with facial vitta between antennal bases not surrounding them; frontal triangle yellow, pale pilose; holoptic, eye bare; vertical triangle black, black pilose; antenna dark brown, basoflagellomere slightly elongate, orangish ventrally; occiput black, silver pollinose, whitish pilose.

*Thorax*: Scutum shiny black, with erected, long yellow pile, white pollinose anteriorly; postpronotum bare; scutellum black, yellow pilose, subscutellar fringe complete with yellow pile. Pleuron black, whitish pilose; metasternum bare; calypter yellow; plumula yellow; halter yellow; spiracular fringes yellow. Wing: Wing membrane hyaline, stigma yellow to dark yellow; extensively microtrichose, bare basally before vein h, costal cell bare on basal 1/6, cell CuP bare very basally, and cell BM bare on basal half. Alula broad, as broad as cell BM, with few microtrichia apically. Legs: Coxae and trochanter black. profemur black on basal 2/5, yellow apically; mesofemur black, yellow on apical 1/4; pro- and meso tibiae yellow with medial dark ring; pro- and mesotarsi brown except basitarsomeres yellow; metafemur black, yellow on apical 1/8; metatibia black, yellow on basal half; metatarsi brown.

*Abdomen*: Petiolate, unmargined. Dorsum mainly black except tergum 2 black with two medial small rounded yellow maculae in the lateral margins; tergum 3 black with two lateromedial small yellow maculae; tergum 4 black with two lateral larger yellow maculae close to anterior margin.

##### Female

Similar to male, with frons shiny black, yellow laterally; yellow maculae of tergum 4 a bit larger.

*Size*: Length: body, 11.0-11.4 mm; wing, 7.1-7.5 mm (male); body, 9.3-10.0 mm; wing, 6.6-7.2 mm (female).

## Discussion

According to the literature, *P. nigricollis* can be found throughout the year, but maybe not so in more tropical regions. Thus it seems plausible to conclude that the species breeds in several generations each year. However, there are insufficient records to draw any firm conclusions. One specimen from Cairo was collected as an adult fly in November (Becker 1903) and one as a larva in 'spring' (Efflatoun 1922). Records from Israel are spread over the year: April, May, June, September and November (Kassebeer 2000). In Cyprus, the only records so far are from the month of November and from one locality only; searches for flies in May 2015 at the collecting site were unproductive.

In the reed beds near Polis where *P. nigricollis* was observed in 2014 and 2015, colonies of Hyalopterus cf. pruni were present on the leaves in May 2015 (Figs 3, 4) but these were not found on 14 November 2015. Up to now no larvae of *P. nigricollis* have been found, only adults in November, some of which were observed mating (Fig. 7). There were no observations of females laying eggs.

The search for larvae needs to be intensified. The presence of aphids on the reeds in May might be the reason for the adults of *P. nigricollis* being found there in November, but this needs to be confirmed. In addition, the relationship with *H. pruni* needs confirmation. After the observed matings in November, it is reasonable to suggest that, at least in Cyprus, *P. nigricollis* lay their eggs shortly afterwards and that the eggs pass the winter in the *Phragmites* vegetation. Furthermore, it is still an open question where to find *Pseudodoros* flies during the rest of the year​.

The occurrence of *P. nigricollis* in Israel and the present discovery in Cyprus, suggests that the range of this species might be approaching Turkey and Greece. The Turkish coast is only 70 km away. The widespread presence throughout Europe of mealy plum aphid (on which the larvae of *P. nigricollis* predate), would suggest that this syrphid species could be expected northward in Europe.

## Supplementary Material

XML Treatment for Pseudodoros (Pseudodoros) nigricollis
